# Taste in Art—Exposure to Histological Stains Shapes Abstract Art Preferences

**DOI:** 10.1177/2041669517736073

**Published:** 2017-10-13

**Authors:** Antonia M. Böthig, Gregor U. Hayn-Leichsenring

**Affiliations:** Psychology of Beauty Group, Institute of Anatomy I, Jena University Hospital, Germany

**Keywords:** artwork, mere exposure, taste, preference, histology

## Abstract

Exposure to art increases the appreciation of artworks. Here, we showed that this effect is domain independent. After viewing images of *histological stains* in a lecture, ratings increased for restricted subsets of abstract art images. In contrast, a lecture on *art history* generally enhanced ratings for all art images presented, while a lecture on *town history* without any visual stimuli did not increase the ratings. Therefore, we found a domain-independent exposure effect of images of histological stains to particular abstract paintings. This finding suggests that the ‘taste’ for abstract art is altered by visual impressions that are presented outside of an artistic context.

Especially in art appreciation, our experiences shape our perception and appreciation of objects. Previously, it has been shown that fluent titles (Gerger & Leder, 2015), successful interpretation (Russell, 2003) and expertise (Belke, Leder, & Carbon, 2015) can contribute to a positive aesthetic experience with abstract artworks. Additionally, the *mere exposure effect* implies that repeated confrontation with a stimulus increases its appeal (Zajonc, 2001). The effect is observed for the preference ratings of art portraits (Belke, et al., 2015) and other artistic paintings (Cutting, 2003). Therefore, confrontation with images of artworks increases ratings on liking of artworks. Furthermore, a domain independence of the mere exposure effect has been described for nonart stimuli (Monahan, Murphy, & Zajonc, 2000). Following this finding, we asked whether confrontation with certain nonart stimuli leads to an increase in liking of abstract artworks as well.

We hypothesised that images of stained histological sections, such as used in medical training and diagnostic procedures (Figure 2(a) to (c)), might influence taste in abstract art because, although they are not intentionally designed to be aesthetic, they appear to be aesthetically pleasing and are similar to certain abstract paintings with regard to subjective appearance and statistical image properties (SIPs). In order to test whether information on and exposure to histological images have an impact on the preference for specific artworks (*stimulus-specific effect*), we asked participants to rate the same set of heterogeneous abstract art paintings before and after lectures on three different topics (*abstract art*, *histology* and *town history*) and compared the ratings (see Figure 1 for experimental design). A lecture on the development of different *abstract art* styles (*art history* lecture) functioned as positive control because exposure to and information on art increase pleasure ratings (*content-specific effect*, e.g., Stojilovic & Markovic, 2014). As a negative control, we gave a lecture on *town history* without any visual stimuli.

**Figure 1.**
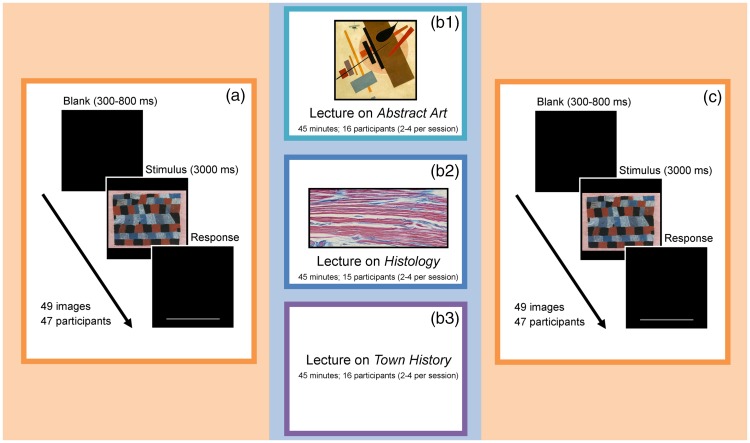
(a) Participants (mean age = 19.7, 40 female) rated images of abstract paintings on a continuous-looking scale ranging from 0 to 1 (100 steps). (b1-b3) Next, the participants were randomly assigned to three different lectures. Group 1 received a lecture on *art history*, (example image from the *art history* lecture: Kazimir Malevich: ‘Supremus No. 58’ (1916)) which included the exposure to 28 images of abstract art paintings (different from the previously rated artworks). Group 2 received a lecture on *histology* including 41 images of histological specimens (see Figure 2). Group 3 heard a lecture on *town history* without any visual stimuli. (c) Afterwards, participants were asked to rate the images of abstract paintings again.

**Figure 2.**
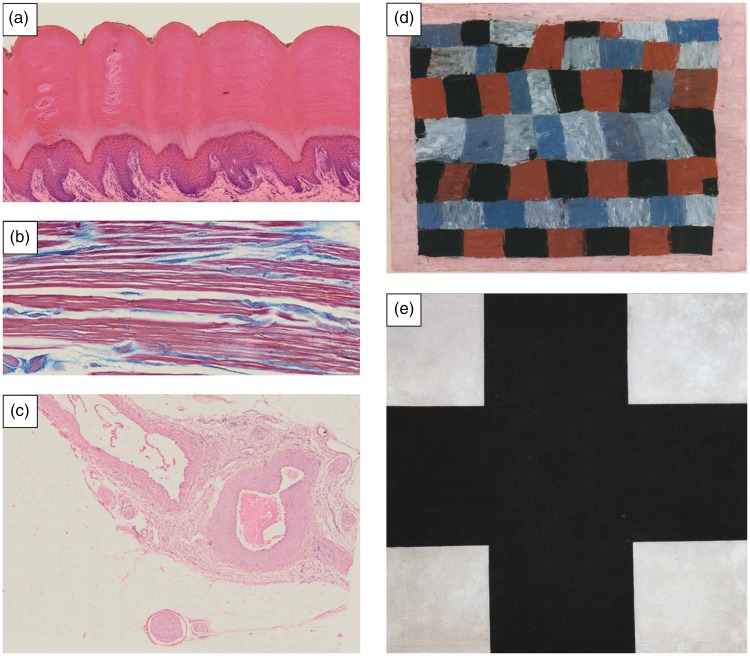
Examples of histological specimens: (a) digital pulp, (b) skeletal muscle and (c) blood vessels (images by Torsten Bölke and Andreas Gebert, Jena University Hospital). Examples of abstract artworks that have been rated in the experiments: (d) *Constructivism*: Paul Klee ‘Rhythmisches, strenger und freier’ (1930) and (e) *Suprematism*: Kazimir Malevich ‘Black Cross’ (1923).

The rated images were categorised by artistic style (see Figure 2(d) to (e)) and analysed for SIPs in order to test for an influence on art taste (stimulus-specific effect).

We found the highest increase for overall liking after the *art history* lecture followed by the *histology* lecture. Specifically, ratings for nearly all art styles increased after the *art history* lecture. After the *histology* lecture, only *Action Paintings* and *Colour Field Paintings* (the subsections of *Abstract Expressionism*) showed increase in rating. Images from none of the styles were rated better after the *history* lecture (Figure 3). Instead, ratings decreased for some art styles (probably due to boredom of the participants). Furthermore, for the *histology* lecture, there was a positive correlation between rating differences and higher values in self-similarity, as well as lower values in anisotropy of the images (Table 1) . Additionally, more complex images were less appreciated after the *history* lecture.

**Figure 3.**
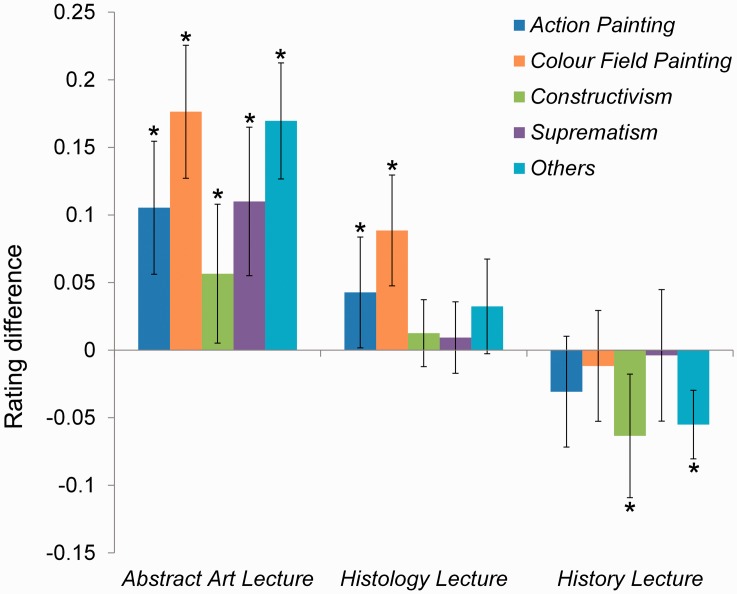
The rated images of abstract art paintings were categorised for art style. Displayed are the absolute rating differences (1 = full scale) for each group, separated by art style. Bars represent confidence intervals (95%). *p < .05.

**Table 1.** SIPs for the Rated Images of Abstract Artworks (Braun, Amirshahi, Denzler, & Redies, 2013).

	HOG complexity	PHOG self-similarity	HOG anisotropy	Aspect ratio	Colour hue	Colour saturation	Colour value
Rating difference for *Art History* lecture (Group 1)	−0.185	−0.076	−0.076	−0.048	0.079	0.113	0.08
Rating Difference for * Histology* lecture (Group 2)	−0.072	.284*	−.360*	0.054	0.039	0.079	0.20
Rating difference for * History* lecture (Group 3)	−.312*	−0.261	0.121	−0.175	0.086	−0.011	0.18

*Note.* Displayed are the correlations between rating differences (before and after the respective lectures) and SIPs. SIPs: statistical image properties; HOG: Histogram of Oriented Gradients; PHOG: Pyramidal HOG.

* *p* < .05.

We confirmed that exposure to and information on abstract art increase its appreciation (Cutting, 2003). Interestingly, the shown effect is domain independent for nonart images on images of abstract artworks. The effect was (a) style specific and (b) sensitive for certain SIPs.

**Table 2.** Mean Values and Standard Deviations of Selected SIPs for the Different Art Styles (Rating Experiment) and Images Used in the Lectures.

	HOG complexity	HOG anisotropy	PHOG self-similarity
*Action Paintings*, *n* = 12	12.67 ± 8.61	0.000465 ± 0.000183	0.72 ± 0.13
*Colour Field paintings*, *n* = 11	5.24 ± 2.83	0.000730 ± 0.000328	0.67 ± 0.10
*Constructivism*, *n* = 10	8.55 ± 4.19	0.000710 ± 0.000252	0.71 ± 0.11
*Suprematism*, *n* = 4	5.15 ± 1.55	0.000971 ± 0.000302	0.62 ± 0.08
*Other*, *n* = 12	9.83 ± 5.39	0.000530 ± 0.000208	0.68 ± 0.15
*Art history* lecture, *n* = 28	13.64 ± 11.35	0.000801 ± 0.000530	0.65 ± 0.19
*Histology* lecture, *n* = 41	12.662 ± 6.19	0.000416 ± 0.000200	0.75 ± 0.12

*Note.* SIPs: statistical image properties; HOG: Histogram of Oriented Gradients; PHOG: Pyramidal HOG.

1. Ratings for images from *Abstract Expressionism* increased after the *histology* lecture. Subjectively, there is a similarity between histological images and these particular art styles. Both are very colourful and organic. Possibly, the participant’s receptivity for such patterns was enhanced after the *histology* lecture.
2. Participants ratings increased for objectively more self-similar and more isotropic images after the *histology* lecture. Generally, artists create images with rather high values in self-similarity (Graham & Redies, 2010). Similar values can be found in natural scenes (Olshausen & Field, 1996) and, interestingly, in histological images (Table 2). Therefore, histological images may sensitise the observer for Self-similarity. Furthermore, histological images are highly isotropic. Subsequently, a familiarity effect would explain the shift in rating preference towards more isotropic images of abstract artworks.

In total, our study provides evidence that information on and exposure to histological stains (aesthetic natural patterns) have an influence on rating preferences on images of abstract artworks. Therefore, nonartistic stimuli can alter personal taste in art.
